# Non-coding RNA in tumor-infiltrating regulatory T cells formation and associated immunotherapy

**DOI:** 10.3389/fimmu.2023.1228331

**Published:** 2023-08-21

**Authors:** Yue Ma, Xin Xu, Huaitao Wang, Yang Liu, Haiyan Piao

**Affiliations:** ^1^ Department of Gynecology, Cancer Hospital of Dalian University of Technology (Liaoning Cancer Hospital & Institute), Shenyang, Liaoning, China; ^2^ Department of Clinical Epidemiology, Shengjing Hospital of China Medical University, Shenyang, Liaoning, China; ^3^ Department of General Surgery, Shengjing Hospital of China Medical University, Shenyang, Liaoning, China; ^4^ Department of Oncology, Shengjing Hospital of China Medical University, Shenyang, Liaoning, China; ^5^ Medical Oncology Department of Gastrointestinal Cancer, Cancer Hospital of Dalian University of Technology (Liaoning Cancer Hospital & Institute), Shenyang, Liaoning, China

**Keywords:** Tregs, miRNA, lncRNA, immunotherapy, cancer

## Abstract

Cancer immunotherapy has exhibited promising antitumor effects in various tumors. Infiltrated regulatory T cells (Tregs) in the tumor microenvironment (TME) restrict protective immune surveillance, impede effective antitumor immune responses, and contribute to the formation of an immunosuppressive microenvironment. Selective depletion or functional attenuation of tumor-infiltrating Tregs, while eliciting effective T-cell responses, represents a potential approach for anti-tumor immunity. Furthermore, it does not disrupt the Treg-dependent immune homeostasis in healthy organs and does not induce autoimmunity. Yet, the shared cell surface molecules and signaling pathways between Tregs and multiple immune cell types pose challenges in this process. Noncoding RNAs (ncRNAs), including microRNAs (miRNAs) and long noncoding RNAs (lncRNAs), regulate both cancer and immune cells and thus can potentially improve antitumor responses. Here, we review recent advances in research of tumor-infiltrating Tregs, with a focus on the functional roles of immune checkpoint and inhibitory Tregs receptors and the regulatory mechanisms of ncRNAs in Treg plasticity and functionality.

## 1 Introduction

Immunotherapy for cancer has become the most promising approach in recent years (1). Therapeutic drugs and cellular therapies derived from immunotherapy have extended the lives of patients with advanced cancer. Among them, immune checkpoint inhibitors (ICIs), also known as co-inhibitory receptors (CIRs), are the most well-known treatment strategy. The expression of ICIs in T cells increases upon activation, forcing the anti-tumor T cells to retreat into a dormant or exhausted non-active state. Antibodies antagonizing ICIs are used to revive T cells and maintain their anti-tumor response (2). So far, immune checkpoint blockade (ICB) antibodies have shown success in some cancers. However, only partial responses or no response have been observed in 60%–70% of patients undergoing this therapy. Additionally, some patients experience immune-related adverse events (irAEs), including autoimmune diseases or immunopathological disorders (3). Therefore, the critical focus of immunotherapy is to identify the reasons behind the shortcomings of ICB treatment and explore more effective anti-tumor immune therapeutic approaches. It will help patients avoid excessive exposure to irAEs and achieve more positive treatment outcomes.

Regulatory T cells (Tregs) are an integral part of the immune system because they contribute primarily to maintaining collective immune system homeostasis and tolerance (4). Tregs inhibit the activation and differentiation of CD4^+^ helper T and CD8^+^ cytotoxic T cells, thereby inducing responses to autologous and tumor-expressed antigens (5, 6). The discovery of suppressor T cells was aided by identifying high and low expression of IL-2 receptor α subunit (CD25) and IL-7 receptor α subunit (CD127) respectively in both humans and mice. The further discovery of forkhead/winged helix transcription factor (Foxp3) expression in Tregs established the CD4^+^CD25^+^Foxp3^+^ classical combination marker (7–9). Adjusted expression of Foxp3 – *via* either a genetic or epigenetic route – significantly affect the immunosuppression of Tregs (10, 11). In the tumor microenvironment (TME), Tregs are majorly immunosuppressive, antagonize anti-tumor immunity and inhibit functions of other immune effector cells, and this aids tumor immune escape (12, 13). Interactions among Tregs, the TME and adjacent cells are critical for maintaining Tregs stability and plasticity. These interactions synergistically influenced the function and number of Tregs through inflammation, cytokine secretion, metabolic changes and transcriptional regulation (14–16). Notably, the infiltration of Tregs into the TME is closely related to tumor progression and poor prognoses (17, 18). However, animal studies have indicated that systemic depletion of Tregs can enhance anti-tumor immunity but can also lead to various autoimmune diseases (19, 20). Therefore, selectively depleting tumor-infiltrating Tregs within the TME without affecting Tregs in healthy tissues can elicit anti-tumor immunity without inducing detrimental autoimmunity. The effective identification of tumor-infiltrating Tregs and a clear understanding of the transition from healthy Tregs to tumor-infiltrating Tregs based on Tregs plasticity represent a promising direction for cancer immunotherapy.

Noncoding RNA (ncRNAs) do not translate proteins (21, 22) but instead act as “regulators” of cellular functions, including molecular signaling pathways in malignant tumors (23, 24). Among them, microRNA (miRNA), long non-coding RNA (lncRNA) and circular RNA (circRNA) are most important to current cancer research (23, 25, 26). They are widely involved in various malignant phenotypes of cancer. LncRNAs exhibit specific expression patterns across various immune cell types, ranging from hematopoietic stem cells (HSCs) to innate and adaptive immune cells in humans and mice. The expression of specific lncRNAs in human and murine immune cell types suggests their evolutionary conservation (27). Immune-specific ncRNAs exert their effects on hematopoietic differentiation through various mechanisms, including acting as ncRNA/protein decoys and functioning as protein scaffolds, transporters, and recruiters in the nucleus and cytoplasm (28). Notably, most immune-specific ncRNAs appear to recruit protein complexes to specific genomic loci, thereby regulating target gene expression at the epigenetic and transcriptional levels and consequently modulating immune cell activity and differentiation in the nucleus.

In this review, we summarize advancements in research of tumor-associated Tregs and highlight relevant interactions between ncRNA and Tregs. On the other hand, we discuss the potential effects of ICB antibodies on Treg-mediated immune suppression in the context of anti-tumor immunity and summarize the role of ncRNAs in this process. A better understanding of ncRNA-mediated tumor immune regulation, especially their role in the regulation of Tregs function, engenders possible insights into cancer immunotherapy.

## 2 The classifications and plasticity of Tregs

Tregs are sub-classified using four criteria, which are governed by plasticity and functional complexity. First, based on their origin, Tregs are divided into thymus Tregs (tTregs) and peripheral Tregs (pTregs) (29). In relation to ncRNA regulation, CD69/miR-155/SOCS-1 axis is a non-redundant key regulator involved in Tregs development and homeostasis (30). Intrathymic miR-181a/b-1 controlled Tregs cell formation by expressing an adequate signaling threshold. The miR-181a/b-1-deficient Treg showed elevated suppressive capacity and was inversely correlated with CTLA-4 protein levels in thymus and peripheral Tregs (31).

Second, based on their degree of activation, Tregs are divided into central Tregs (cTregs) and effector Tregs (eTregs) (32). CTregs, also known as resting or naive Tregs, are the major constituents of peripheral and secondary lymphoid Tregs. CTregs that express CD62L, CCR7 and are phenotypically similar to traditional naive T cells, are important for circulation in lymphoid organs (33). On the other hand, eTregs are mainly found in tissues and organs, with only a small fraction found in secondary lymphoid organs (34). eTregs are antigen-activated Tregs that highly express CD44, ICOS, and KLRG1 and other molecular and tissue-localization-related chemokine receptors, lack CD62L and CCR7 expressions, and can directly contribute to immunosuppression (35). miR-155 targets and reduces CD62L expression in Tregs (36). It is a critical regulatory factor in pregnancy immune adaptation, promoting Treg expansion to achieve pregnancy tolerance and prevent fetal loss (37). miR-744/CD134 mitigates immune rejection by regulating the expression of CD62L and Ki67 (38).

Third, based on their biological characteristics, Tregs are divided into natural Tregs (nTregs) and inducible Tregs (iTregs) (39). nTregs mature in the thymus, maintain immune tolerance, and control inflammatory responses by exerting inhibitory functions through cell-to-cell communication (39, 40). nTregs are generated from CD25^+^ Tregs and Foxp3^lo^ Tregs progenitors through the acquisition of negative and positive selection programs, respectively, with distinct TCR (T-cell receptor) repertoires and transcriptomes (41). In contrast, iTregs are closely related to cancers, such as intratumoral iTregs that act in a tumor antigen-selective manner. They are activated and expanded in the TME when their TCRs specifically respond to autologous tumors and mutated neoantigens (42). Furthermore, the TCR repertoire of intratumoral iTregs significantly overlapped with circulating Tregs and was also able to exhibit specific responses to autologous tumors and mutated neoantigens. This suggests that TCRs derived from tumor antigen-specific Tregs are present in circulation and the TME, as both were sources of tumor-specific TCRs (42).

Fourth, Tregs have been classified based on cell surface markers. These include Th1-like Tregs (T-bet^+^ IFNγ^+-^ Foxp3^+^), Th2-like Tregs (Gata3^+^ IRF4^+^ IL4^+^ Foxp3^+^) and Th17-like Tregs (IL-17^+^ RORγt^+^ Foxp3^+^). Th1-like Tregs are characterized by the expression of T-bet and CXCR3 (43) and inducing the transformation of each other subtype to Th1 is a potential therapeutic approach. Th2-like Tregs mainly express Gata3 and IRF4 and tend to secrete IL-4 and IL-13 (44), are potent immunosuppressors and promote activation through autocrine IL-2. They are found more in tissues than in circulation and ably migrate in response to chemokines in the TME (45). Th17-like Tregs expressed RORγt and shared some phenotypic and functional characteristics with conventional Th17 cells, such as expressing high levels of CCR4 and CCR6 and low levels of CXCR3. However, Th17-like Tregs expressed only low levels of CD161 and were mostly unable to secrete IL-22 and TNF-α but produced IL-17, thereby retaining their inhibitory function (46, 47). miR-17 modulates Tregs function by targeting co-regulators of the Foxp3 transcription factor (48). Furthermore, Foxp3 plays a dual role in controlling the dependency on IL-2 in Tregs. On the one hand, it inhibits IL-2 transcription; on the other hand, it promotes the expression of CD25 (IL-2Ra) (49, 50). Reprogramming T cell-derived extracellular vesicles through IL-2 surface engineering can induce effective anti-cancer effects through miRNA delivery (51).

In summary, Tregs differentiated into subclasses under various stimuli, and there was mutual transformation among the subclasses. Although this demonstrated the plasticity of Tregs, much of this plasticity remains unanswered. Does plasticity depend on the initial heterogeneity of Treg? Is there an inevitability of specific subsets of Treg with specific effects? Tumor-infiltrating Tregs may arise from circulation and tissue residency and be induced by the TME. Distinguishing their phenotypic and functional characteristics from Tregs in healthy tissues based on plasticity and stability is crucial for the development of immunotherapies that target Tregs.

## 3 The ncRNA associated regulatory mechanisms of Tregs in cancer

In most cancers, Tregs are in higher proportions in tumor than normal tissues and infiltrate tumor tissues earlier than effector T cells. Indeed, a high ratio of Treg to CD8^+^ T cells indicated a poor prognosis (52). Furthermore, the abnormal differentiation and distribution of Tregs in cancer patients are affected by altered genetic information, abnormal molecular expression and reprogramming of cellular metabolism (53). These factors either drive Tregs enrichment in the TME, resulting in an immunosuppressive microenvironment, or aid peripheral Tregs in their roles in the formation of pre-metastatic niches (54).

*Helios* gene promoted the preferential differentiation of CD4^+^ naive T cells into Tregs (55). Intratumoral *Helios*- deficient Tregs acquired effector T cell function and induced immune responses by expressing effector cytokines (56). In malignant pleural effusion (MPE) of Non-Small Cell Lung Cancer (NSCLC) patients, decreased miR-4772-3p levels relieved the repression of *Helios*, thereby enhancing the activity of Tregs (57). Widespread miR-146a in Tregs regulated IFNγ-dependent immune responses by targeting STAT1 (58). Similarly, the loss of miR-17-92 in CD4^+^ T led to tumor immune evasion (59). Expression profiling of miRNAs revealed that miRNAs modulated the biological characteristics of Tregs by acting on target genes such as FOXP3, CTLA-4 and GARP (59, 60). The expression of miR-21 affects the balance of Th17/Tregs in GC patients (61). Among them, the Foxp3^+^ Tregs subset has been a focal point of research in recent years. Peripheral Tregs can be further classified into three subtypes based on the expression of CD25 and Foxp3: Fr. I, naive or resting Tregs with the CD45RA^+^CD25^lo^Foxp3^lo^ phenotype; Fr. II, Fr. I differentiates into CD45RA^+^ CD25^hi^Foxp3^hi^ effector Tregs following antigen stimulation; Fr. III, a subset of CD45RA^+^CD25^lo^Foxp3^lo^CD4^+^ T cells that produce pro-inflammatory cytokines but exhibit minimal suppressive activity (62, 63). The enrichment of Fr. III subtype in cancer tissues is associated with a more favorable prognosis compared to the Fr. II subtype (63). NF-κB-mediated miR-34a disrupts the equilibrium Treg/Th17 balance by directly targeting Foxp3 (64). ADAR1 enhanced Treg cell function *via* modulation of the miR-21b/Foxp3 axis (65).

The immunosuppressive function of Tregs is dependent upon high intracellular cAMP concentrations. One of the major metabolic pathways supporting Tregs survival and function was an altered lipid metabolism (66). In relation to ncRNA regulation, miR-142-5p inhibited the expression of cAMP-hydrolyzing enzyme phosphodiesterase-3b (Pde3b) at the post-transcriptional level to modulate immune metabolism, thereby controlling the function of Tregs (67). Furthermore, given the predominance of intratumoral Tregs in glucose uptake, both glycolysis and oxidative phosphorylation contributed to fatty acid synthesis and thus promoted Tregs expansion (68). Glycolytically produced lactate increased Foxp3+ Tregs expression by activating the NF-κB pathway and promoted prostate cancer (PC) invasion through miR-21/TLR8 (69). Under starvation conditions, Foxp3 expression in human Peripheral Blood Mononuclear Cells (PBMCs) was inversely correlated with the expression of miR-31 and miR-155, which may also be potential metabolic-related immunomodulatory tools (67).

Relatedly, lncRNAs were involved in the regulation of Tregs function at the molecular level in breast cancer, the lncRNA SNHG1 competitively bound to miR-448 and reduced expression of IDO, thus inhibited Tregs differentiation, and this hindered immune escape (70). Generally, highly expressed IDO in the TME led to a decrease in tryptophan and accumulation of kynurenine, inhibiting T cell activation and inducing the production of Tregs (71). *In vitro* and *in vivo* assays confirmed that lncRNA Flatr in T cells directly participated in the transcription of Foxp3 as lncRNA Flatr-deficient mice showed delayed induction of Tregs (72). Besides, high expression of ZC3H12D in Tregs and NSCLC influenced patient prognosis through the ZC3H12D-hsa-miR-4443-ENST00000630242 axis (73). The highly-expressed membrane and cytoplasmic-localized lnc-INSR aided Tregs formation of an immunosuppressive microenvironment by inducing aberrant activation of the PI3K/AKT pathway in childhood acute T lymphoblastic leukemia (74). Overexpressed LINC00301 in NSCLC tissue targeted TGF-β and this increased Tregs therein (75).

**Figure 1:**
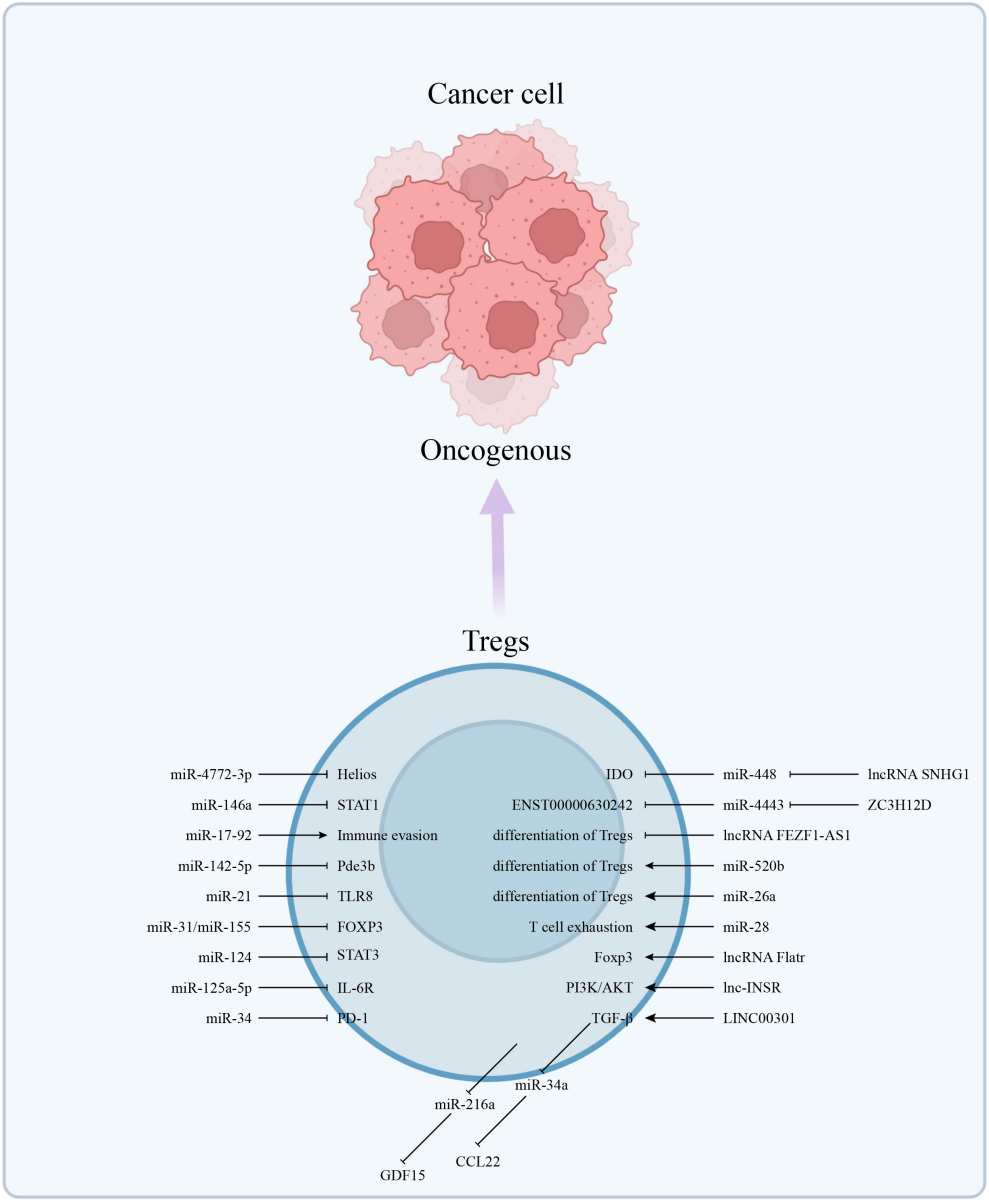
The abnormally expressed ncRNAs in Tregs affect the function of Tregs in multiple dimensions, such as affecting the differentiation of Tregs, the expression of ICIs, and the release of cytokines. Thereby establishing an immunosuppressive microenvironment and promoting the occurrence and development of cancer.

Similarly, miRNAs were important regulators as overexpressed miR-216a was associated with decreased overall survival in CRC, as shown *via* multiomics analysis, and Tregs that had infiltrated the TME were involved in the regulation of miR-216a functions (76). MiR-520b that was overexpressed in breast cancer (BC) tissues augmented activation of Tregs in TME and induced M2-type polarization of macrophages (77). Persistent HBV in hepatocellular carcinoma (HCC) tissue maintained TGF-β activity, which repressed miR-34a expression, which in turn increased both CCL22 and recruitment of Tregs (78). MiR-26a inhibited the HCC-induced effect of diethylnitrosamine (DEN) by reducing the abundance of Tregs (79). MiR-28 was involved in PD1^+^Foxp3^+^expression and influenced the exhaustion of TIM3^+^Foxp3^+^Tregs *in vitro* (80). The GATA3/miR-125a-5p/IL-6R axis explicated how Treg cells responded to inflammatory IL-6-rich conditions (81). Relatedly, MiR-124/STAT3 played a similar role in Tregs of glioma as exposure of T cells of glioblastoma patients to miR-124 reactivated the immune response (82). Mesenchymal stem cells modulated the CRC-TME immunocompetence *via* miR-150 and miR-7 (83). MiR-34 was downregulated in *Tp53*-mutated secondary adult acute myeloid leukemia (sAML), resulting in increased PD-1 expression and Treg enrichment (84) (**Figure 1**).

## 4 The ncRNA and Tregs cell-based communication in TME

### 4.1 Tregs and cancer cells in TME

Signals such as tumor-derived cytokines, exosomes in the TME and tumor antigens work to additionally induce the transformation of T cells into Tregs (85–88). For example, tumor-derived cytokines such as IL-2 and TGF-β are representative molecules that induced Foxp3^+^ Tregs (89, 90) and more on this was summarized by Tuzlak S et al. (91). Furthermore, tumor-derived exosomes, because of their diverse contents, induce Tregs *via* a more complex mechanism (92). First, there exist exosome-lncRNAs that induce Tregs at various levels. For example, CD73^+^γδT1 cells are the predominant Treg subset in breast cancer (BC). Their induction is triggered when lncRNA SNHG16 carried by BC cell-derived exosomes acts as a competing endogenous RNA (ceRNA) by sponging miR-16-5p, leading to the liberation of the target *SMAD5* gene, thereby promoting the expression of CD73 and inducing T cell differentiation into Tregs (93). Another example is lncRNA RP11-323N12.5 that was overexpressed in gastric cancer (GC) and thus activated the Hippo signaling pathway in T cells and induced the differentiation of Tregs through exosome-carrier (94). Likewise, RP11-357H14.17, which was overexpressed in GC, is possibly involved in the differentiation of Tregs (95). One can hypothesize that exosomal miRNAs possibly play a similar role, and this is supported by the uniqueness of miRNAs in Tregs exosomes due to the enrichment of miR-146a-5p, miR-150-5p and miR-21-5p, and depletion of miR-106a-5p, miR-155- 5p and miR-19a-3p (84). Further, colorectal cancers (CRC) secreted miR-208b-containing exosomes by targeting PDCD4 to promote Tregs proliferation and reduce CRC sensitivity to oxaliplatin-based chemotherapy (96). The miR-124-3p-enriched exosomes significantly inhibited CRC growth, reduced Tregs infiltration into the TME, and prolonged the median survival time of tumor-bearing mice (97). Non-Small Cell Lung Cancer (NSCLC) and CRC-derived miR-214 were delivered into T cells *via* microvesicles (MVs), which subsequently downregulated phosphatases and PTEN and promoted Tregs expansion. The miR-214-induced Tregs promoted tumor growth through IL-10. Pertinently, the anti-miR-214 antisense oligonucleotides (ASOs) effectively blocked Tregs expansion and limited tumor growth in tumor-bearing mice (98). Relatedly, nasopharyngeal carcinoma (NPC) exosomal miR-243 targeted FGF11 to inhibit T cell proliferation and induce Tregs to not only differentiate, but also impair T cell function (99). Lastly, miR-10a-loaded exosomes resulted in increased expression levels of RORγt and Foxp3 in T cells that promoted Tregs differentiation (100).

### 4.2 Tregs and other immune cells in TME

**Figure 2:**
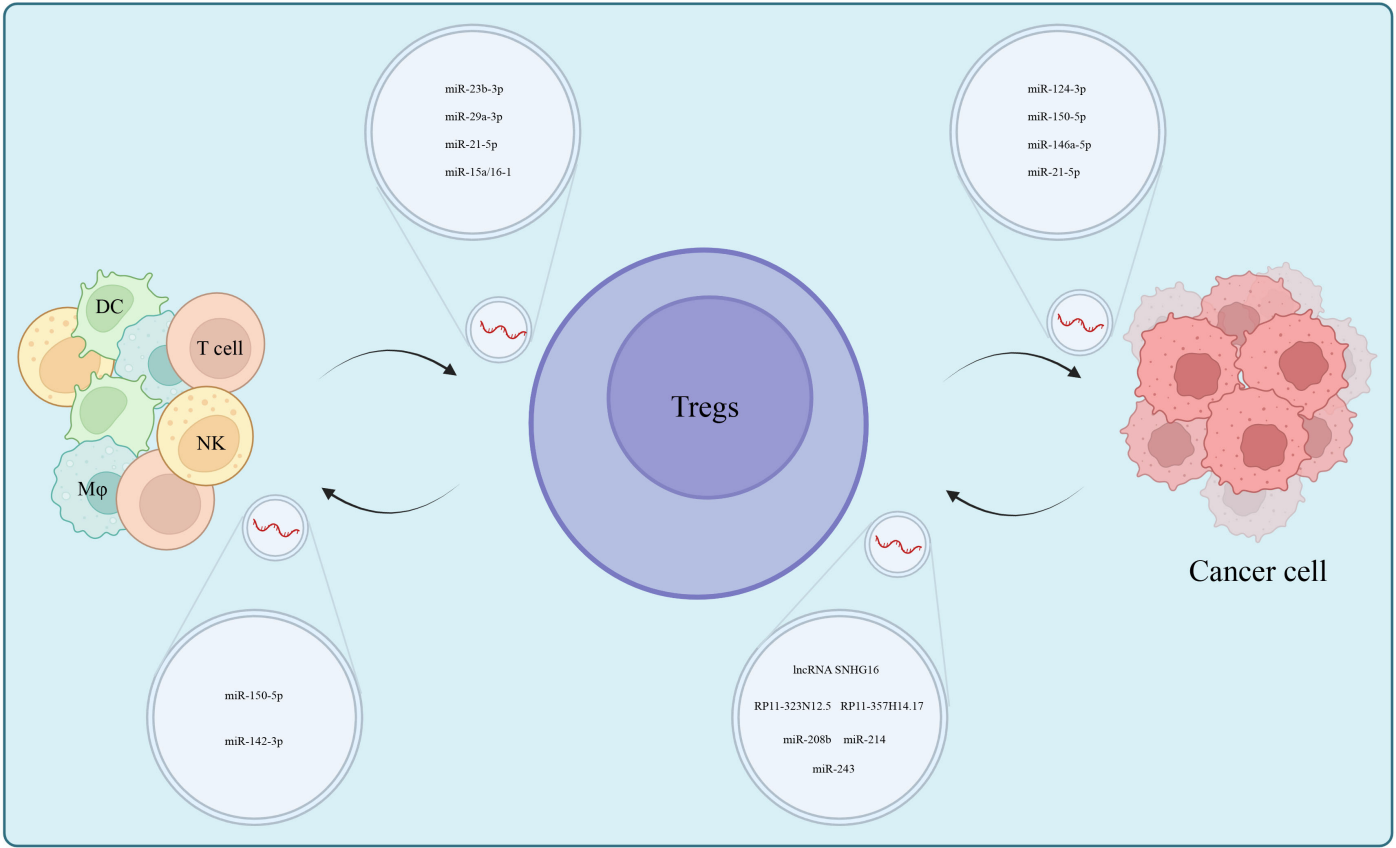
Exosomal ncRNAs mediate cell-to-cell interactions which centered on Tregs in the TME, participate in tumorigenesis and form an immunosuppressive microenvironment.

Differentiated Tregs suppressed the antitumor immunity of effector T cells, NK cells, macrophages and DCs through multiple mechanisms, and functioned synergistically with MDSCs through crosstalk (101). Generally, Tregs inhibit antitumor immune functions of DCs cells by secreting inhibitory cytokines (IL-10, TGF-β and IL-35) (102, 103). In addition, CTLA-4 and LAG3 on the surface of Tregs combined with CD80/CD86 and MHC II, respectively, on the surface of DCs to induce immune tolerance of DCs (104, 105) suggesting that cell-to-cell transfer of ncRNAs *via* exosomes might be a novel mechanism by which Tregs regulated DCs function. Indeed miR-150-5p and miR-142-3p, upon entry into DCs, promoted an increase of IL-10 and a decrease of IL-6, which suppressed immune response in tissues (106). DCs cells influence the differentiation of Tregs as TGF-β selectively increased the expression of miR-27a in DCs through transcription factor SP1, and this hindered DC-mediated Th1 and Th17 cell differentiation but promoted Tregs differentiation (107). Akin to this was that overexpressed CTLA-4 in RORγt-deficient Treg that were isolated from tumors, increased Foxp3 expression in DCs cells (108). Exosomal miR-29a-3p and miR-21-5p released by macrophages in the epithelial ovarian cancer (EOC) microenvironment synergistically inhibited STAT3, resulting in an imbalanced Treg/Th17 ratio, which created an immunosuppressive microenvironment (109). Conversely, miR-15a/16-1 alleviated immunosuppression in HCC by disrupting CCL22-mediated communication between Kupffer cells and Tregs (110). The positive feedback loop formed between MDSCs and Tregs contributes to the formation of the immunosuppressive microenvironment. Tumor-induced MDSCs promoted the proliferation of Tregs in both a TGF-β-dependent and highly expressed CD73 manner, enhancing immunosuppressive effects (111, 112). Then, Tregs enhanced the expansion and suppressive functions of MDSCs by promoting the secretion of TGF-β and IL-35 (113, 114). MDSCs- and Tregs-associated miRNAs have been identified in acute lymphoblastic leukemia (115). Of note is that BMSCs-derived exosomal miR-23b-3p maintained Th17/Treg balance, by suppressing the PI3K/AKT/NF-κB signaling pathway (116) (**Figure 2**).

### 4.3 Tregs in circulation

NcRNAs in circulation are involved in the regulation of Tregs. MiR-21 expression was significantly elevated in the serum of diffuse large B-cell lymphoma (DLBCL) patients, and it promoted inducible co-stimulator (ICOS) expression on Tregs, thereby assisting the COS ligand (ICOSL) on endothelial cells to crosstalk with Tregs (117). The upregulated miR-21 was also associated with the different subsets of Th cells in esophageal squamous cell carcinoma (ESCC) (118). Overexpressed miR-182 in peripheral blood mononuclear cells (PBMCs) and serum from BC patients increased the expression of FOXP3, TGF-β and IL-17 in T cells and induced T cell differentiation into Tregs (119). Similarly, the upregulated linc-POU3F3 in PBMCs recruited TGF-β, increased the phosphorylation level of SMAD2/3, and ultimately promoted the distribution of Tregs among peripheral blood T cells of GC patients (120). MiR-27b-3p, miR-340-5p and miR-330-3p negatively regulated TGF-β and IL-10 in CD8+ T cells, limiting their differentiation into Tregs (121). Overexpression of miR-155 in peripheral blood and tissues of cervical cancer patients (CC) inhibited the expression of the target *SOSC1* gene and induced an imbalanced Th17/Treg ratio (122). MiR-141 targeted CXCL1/CXCR2, which reduced Tregs recruitment in MPEs of NSCLC (123). MiR-568 mimiced its target NFAT5, thus suppressing Tregs cell activation and reducing Treg-derived IL-2 production (124). On the other hand, the differentiated Tregs regulated, *via* positive feedbacks, tumor development and immune evasion. Lnc-EGFR was overexpressed in Tregs and promoted HCC growth in an EGFR-dependent manner (125).

Many questions on circulating ncRNAs remain unanswered. Are they delivered in the form of exosomes or microvesicles, or are they purely independent RNA molecules? If they are free circulating molecules, how do they overcome blood flow shear stresses and avoid enzymatic degradation? Whatever the case, this long-distance signaling is the basis for the formation of pre-metastatic niches and molecular targets in liquid biopsies.

### 4.4 The role of immune-related ncRNA in prognostic prediction models for cancer

Given the broad regulatory roles of ncRNAs in the immunosuppressive microenvironment, their aberrant expression is possibly related to tumor prognosis. Indeed, the predictive models based on immune-related ncRNA expression contributed to evaluating the prognosis of head and neck squamous cell carcinoma (HNSCC) (126), GC (127), pancreatic cancer (128) and hepatocellular carcinoma (HCC) (129). MiR-146a expression in PBMCs was not only negatively correlated with Tregs but was also a marker of NSCLC liquid biopsy (130). The expression of miR-21 was associated with the inhibition of CD8^+^ T and was a potential diagnostic and prognostic marker for ESCC (131). The combined expression of miR-101, miR-548b, miR-554, and miR-1202 was a prognostic marker and potential therapeutic target for PCNSL (132). FGD5-AS1 promotes apoptosis of CD8^+^ T by influencing the expression of PD-L1 in NSCLC cells and was associated with poor prognosis of patients (133). The exosome circUHRF1 secreted by HCC cells contributes to immunosuppressive by inducing NK cell dysfunction and leading to adverse clinical outcomes (134).

## 5 Tregs-related tumor immunotherapy strategies

**Figure 3:**
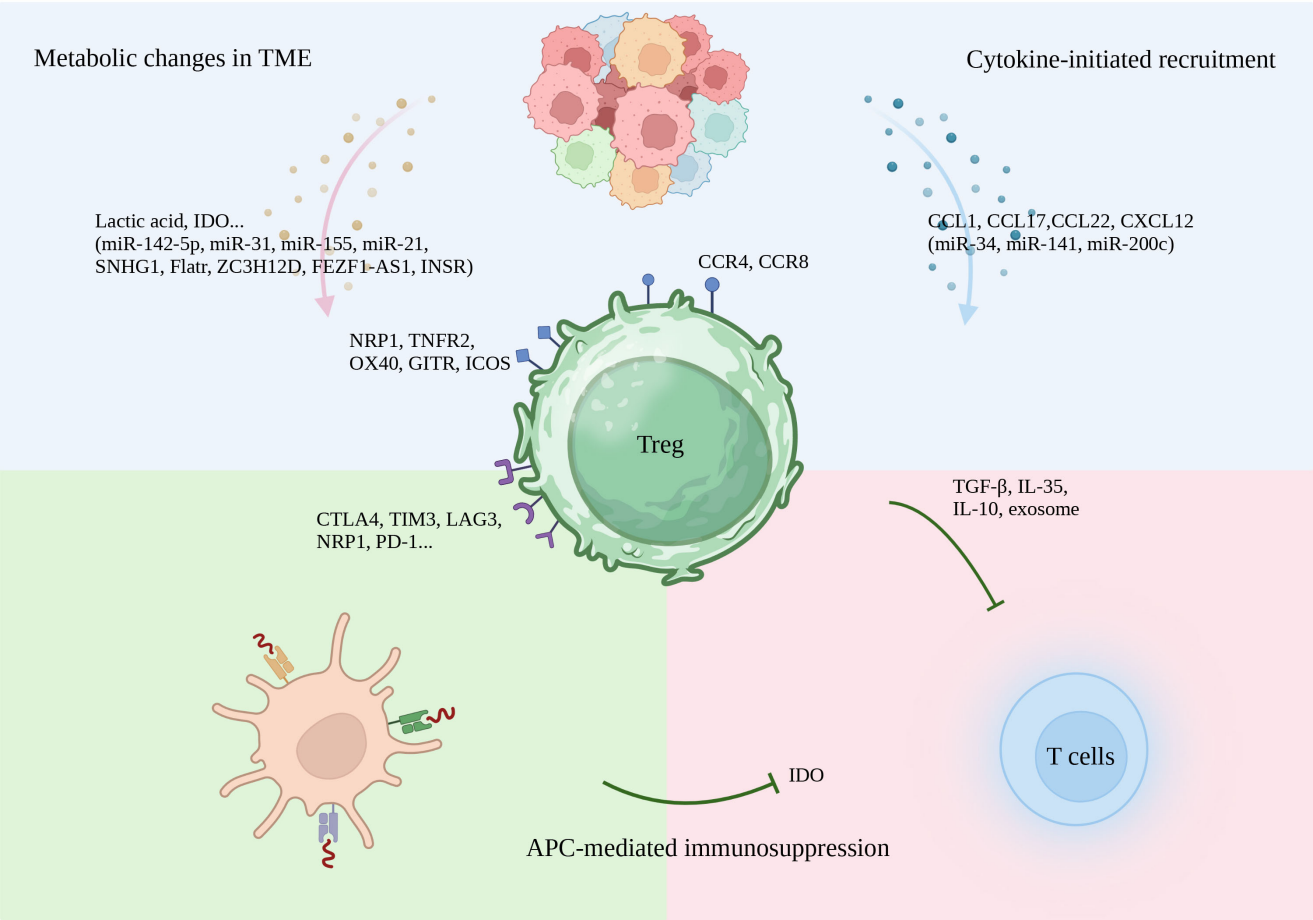
Major immunosuppressive functions of Tregs.

The TME has an elevated ratio of Tregs to effector T cells, which effectively suppresses autologous antitumor immune responses (**Figure 3**). Thus, reducing the infiltration of Tregs into the TME reverses this immunosuppression (135). This can be attained through either depletion of Tregs or reducing recruitment into the TME. Other ways include taking advantage of the plasticity of Tregs to transform them into an anti-tumor phenotype and, finally, applying ICIs therapy to change the biological behavior of Tregs.

### 5.1 Depletion and reduced recruitment of Tregs

Blockades of Tregs by CD25 restored IFN-γ production in CD8^+^ T cells and improved the efficacy of anti-VEGF therapy, which extended relapse-free survival durations in glioblastoma patients (136). Blocking IL-2 signaling —the use of anti-IL-2/anti-IL-2R to do so improved immune responses— impaired Tregs activity. Indeed, treatments with IL-2 mutant proteins reduced the number of Tregs and inhibited tumor growth (137). In addition, miR-142-3p in Tregs mediated cyclophosphamide (CY) depletion of Tregs by both targeting CD39 and altering intracellular ATP levels (138). Exogenous supplementation of miR-200c combined with the B16F10/GPI-IL-21 vaccine reduced Tregs recruitment, activated antitumor immunity and reduced melanoma metastasis (139). While depletion of Tregs appears to be a plausible therapeutic direction, Tregs that infiltrated TMEs lacked unique identifiers, thus creating a risk of clearing non-target Tregs, which may cause unexpected physiological damage. More insights are thus needed to make such treatments clinically feasible. Another promising therapeutic strategy is blocking the migration of Tregs into the TME by restricting intercellular communication that is based on CCR4 (140), CCL20 (141), CCL3-CCR1/CCR5 and CXCL12-CXCR4 (142). Relatedly, radiotherapy inhibited the specific recruitment of Tregs in Lewis lung cancer by upregulating miR-545 (143). A worthwhile research focus is the application of ncRNA for similar therapeutics.

### 5.2 Predisposing Tregs to an antitumor immunophenotype

This can be attained through first, curbing the transformation of CD4^+^T to Tregs —miR-17-92 (59, 144) is involved in the differentiation of CD4^+^ into Tregs— and second, inducing the transformation of Tregs to Th1 type —Th1-like differentiation was mediated by miR-27a (107). In addition, ncRNAs function as transit points in the drug-mediated transformation of Tregs. Shenmai injection inhibited the differentiation of CD4^+^ T cells into Tregs through the miR-103/GPER1 axis, thereby improving postoperative immune function in patients with papillary thyroid carcinoma (PTC) after 131 I radiotherapy (145). *Ganoderma lucidum* polysaccharides (GLPS) increased miR-125b expression, which then repressed Notch1 and Foxp3, restoring T cell function and limiting HCC growth (146). It is worth emphasizing that the oncogenic and tumor suppressor function of the Th17 subtype of Tregs in the TME is currently not fully understood. This together with the concomitant complex molecular regulation is a worthwhile research topic.

### 5.3 The role of ICIs in Tregs in cancer

**Figure 4:**
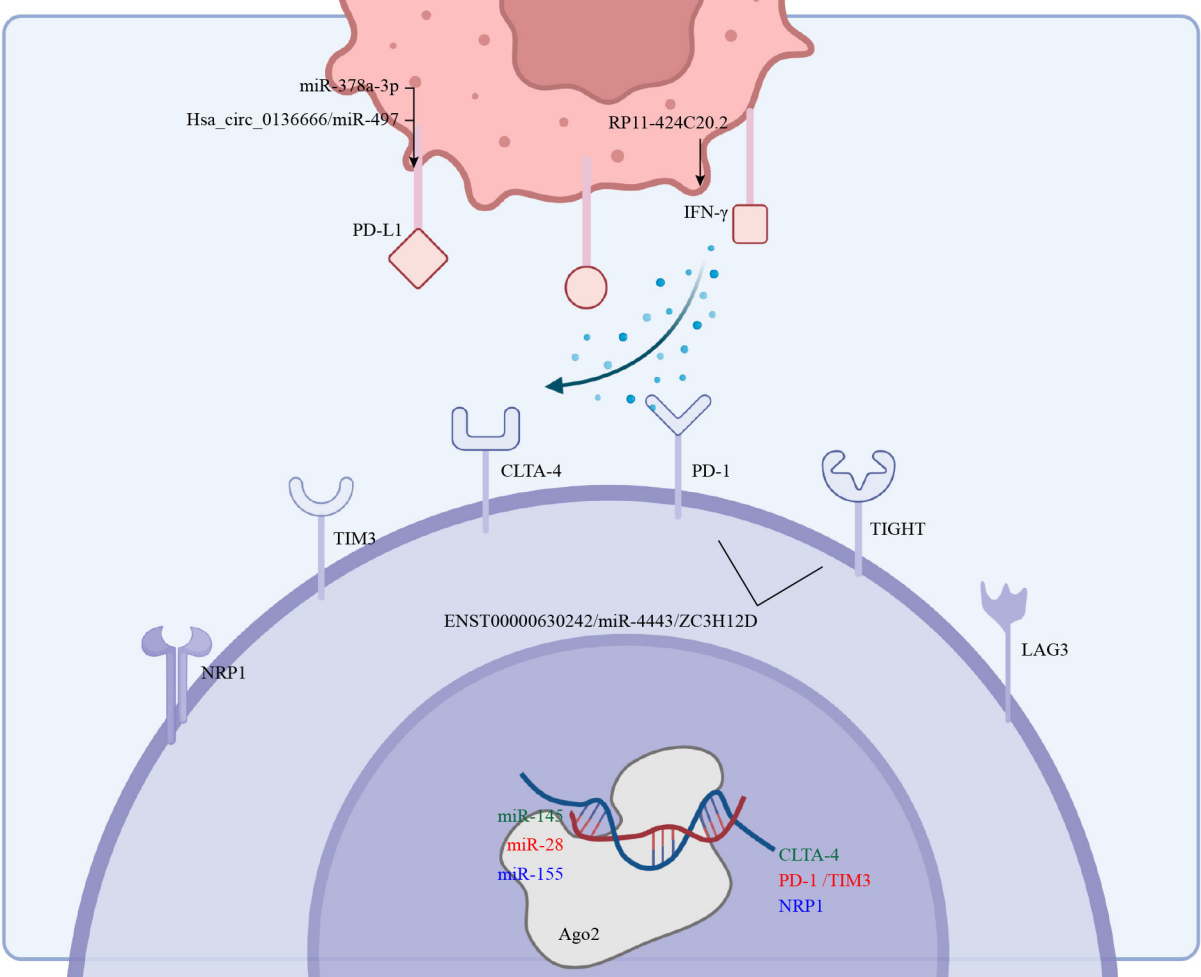
The ncRNAs in cancer cells and Tregs affect the expression of ICIs and ligands which induce immunotherapy resistance.

Altered expression of ICIs underlies cancer evasion from immune surveillance (147), and immunotherapies that targeted CTLA-4 and PD-1 were effective against a variety of cancers (148). Indeed, targeting CTLA-4, TIGIT, PD-1, GITR and other co-inhibitory receptors to limit the function of Tregs possibly is an effective cancer treatment (149). Although tumor-infiltrating Tregs are functionally conserved, and tumor-infiltrating effector T cells are dysfunctional, co-suppressor receptors do not have opposing effects on Tregs and effector T cells. Much remains to be clarified on the effects of either checkpoint inhibition or stimulation not only on Tregs stability and function but also on Tregs and effector T cell activity and ratio (**Figure 4**).

#### 5.3.1 CTLA-4

CTLA-4 was the first identified immune checkpoint of Tregs that inhibited functions of CD4+ and CD8+ cells (150), and part of this inhibition was achieved through high-affinity binding to CD80 and CD86 (151). Abnormally expressed ncRNAs in Tregs were also an important factor in regulating CTLA-4 expression (108), as miR-145 negatively regulated the expression of CTLA-4 by binding to its 3’-UTR in Tregs (59). Thus, anti-CTLA-4 therapy could enhance the functions of effector T cells, only after surmounting these two challenges. First, anti-CTLA-4 therapies are yet to achieve the same effects as the current benchmark, anti-PD-1 therapies. Indeed, although anti-CTLA-4 mAbs were effective in depleting Foxp3+ Tregs in mouse tumors, similar success has not been attained in human tumors (151) because anti-CTLA-4 mAbs depleted Tregs in an Fc-dependent mechanism. Thus, antibodies with improved FcγR binding profiles were able to induce better intratumoral Tregs depletion and increased CD8+/Tregs ratio (152). This is possibly due to high antibody-dependent cell-mediated cytotoxicity (ADCC) and cellular phagocytosis (ADCP) (153, 154). On the other hand, treatment with anti-CTLA-4 mAb combined with IL15/IL15Rα complex depleted Tregs, which might have been related to the activation of NK cells (155). Yet even low-doses of anti-CTLA-4 combined with anti-PD-1 therapy led to immunotherapy-related adverse effects (irAEs) (156). Fortunately, recent results showed that the introduction of a tyrosine-to-histidine mutation in the polypeptide chain improved the pH sensitivity of anti-CTLA-4 mAb, thereby avoiding CTLA-4 downregulation and effectively depleting intratumoral Tregs, which then reduced occurrences of irAEs (157). Thus, either TLA-4 and OX40 bispecific antibodies or EZH2-based anti-CTLA-4 therapy are putative next-generation immunotherapies (158). Lastly, it was suggested that the expression of CTLA-4 maintains the balance between effector T cells and Tregs and adjusting its expression through RNA-related technologies was a therapy likely to destroy the immunosuppressive TME (159). The RP11-424C20.2/UHRF1 axis in HCC and thymoma affects CLTA-4 expression in an IFN-γ-dependent manner (160).

#### 5.3.2 PD-1/PD-L1

Through binding with its ligand (PD-L1 or PD-L2) on the surface of Tregs, PD-1 restricted activation of the PI3K/AKT/mTOR pathway by dephosphorylating CD28 (161, 162). Further, exogenous TGF-β and PD-L1 induced T cells to differentiate into Tregs (163). No wonder anti-PD-1/PD-L1 therapy is patently the most effective current anti-tumor immunotherapy in clinical practice (164). In terms of ncRNA regulation, miR-378a-3p affected Tregs differentiation by directly regulating PD-L1 in HCC (165). Similarly, aberrantly activated Hsa_circ_0136666/miR-497/PD-L1 axis in CRC regulated Treg-mediated immune escape in a similar manner (166).

#### 5.3.3 TIGIT

TIGIT competes with CD155 on the cell surface, and this influences the phenotypic variation of Tregs (167). TIGIT is transcriptionally regulated by FOXP3 and is therefore regarded as an identifier of pure and stable Tregs (168, 169). TIGIT^+^ Tregs were more effective at suppressing TH1 and TH17 cell responses than TIGIT^-^ Tregs. Functionally, TIGIT works by inhibiting the PI3K–AKT pathway (170). Notably, the enrichment of TIGIT^+^ Tregs has been detected in various cancer groups (171, 172). In terms of ncRNA regulation, ENST00000630242 (lncRNA) was involved in the expression of TIGIT in NSCLC through a ceRNA mechanism (73).

#### 5.3.4 LAG3

LAG3 is a co-inhibitory receptor that acts both intracellularly and extracellularly in Tregs cells. It is required for Tregs cell-mediated suppression of effector T cell proliferation (173, 174). On the other hand, LAG3 is highly expressed on IFNγ+FOXP3+ TH1-like Tregs and may be a potential marker of TH1-like Tregs. Similarly, the prevalence of LAG3^+^Treg in peripheral blood was higher in cancer patients than in healthy volunteers, but altogether lower than in the TME (175, 176). However, like CTLA-4, LAG3 enabled Tregs proliferation and limited Treg accumulation at sites of inflammation (104, 177). Moreover, the expression of LAG3 was proportional to that of CD25, which counteracted both activation of Treg cells and specific upregulation of LAG3-LAG3 functions in Treg are complex (177).

#### 5.3.5 TIM3

TIM3 is not only highly expressed in tissue-Tregs but also often co-expressed with other inhibitory receptors (178). Infiltration of TIM3^+^Tregs into the TME of various cancers preceded that of the peripheral blood (179). Furthermore, TIM3^+^Tregs were a resident Tregs subset in colon cancer (180). In relation to ncRNA regulation, overexpressed miR-28 in melanoma-bearing mice inhibited the expression of PD-1 and TIM3 on Tregs and induced the depletion of TIM3^+^ Foxp3^+^ Tregs (80).

#### 5.3.6 NRP1

NRP1 expressed by Tregs was an important molecule in the discrimination between thymus- and peripherally-derived Tregs (181). It was specifically expressed in immunosuppressive environments such as cancers and was a potential VEGF receptor (182). The expression of NRP1 promoted not only interactions between Tregs and DCs but also peripheral immune tolerance (183, 184). In relation to ncRNA regulation, deficiency of miR-155 impeded NRP1-mediated immune tolerance (185).

## 6 Conclusions and perspectives

Tregs are a key mediator of immune self-tolerance, which in turn facilitates autoimmunity and tumor immunosuppression. Moreover, Tregs are a highly plastic and heterogeneous cell population. Their immunosuppressive effects are a major obstacle to effective antitumor immunity. Thus, augmenting traditional cancer treatment methods with therapies that target these immunosuppressive effects can create effective anti-tumor effects that are especially relevant in the field of immunotherapy. The complex functions of intratumoral Tregs are affected by both exogenous and endogenous factors. Important endogenous factors are abnormal transcription and reprogrammed metabolism, whereas important exogenous factors are cytokines, chemokines, exosome contents, and metabolites in TME. Various ncRNAs patently play powerful roles in inducing the differentiation of Tregs and regulating the expression of ICIs. Thus, a possible strategy for improving cancer treatments is using immunotherapies that target ncRNA. Indeed, clinical trials of ncRNA-focused tumor therapies are ongoing (NCT03830619, NCT04269746, NCT04767750, NCT03057171), yet not one study has validated ncRNAs as targets for regulating Tregs in cancer immunotherapy. Exacerbating this dearth of research on ncRNAs-targeting therapies is the few studies on ncRNAs in Tregs —even among these few studies, the majority are on autoimmune diseases when the most impactful focal areas for cancer research would be to not only understand the role of ncRNAs in tumor-infiltrating Tregs and but also construct corresponding regulatory networks.

Tumor immunotherapy that targets Tregs remains both promising and challenging. For example, Tregs-related therapy has shifted from “elimination” to “inducing the functional differentiation of Tregs towards Th1-like Tregs”. Thus, exploring targets in this new direction, especially those related to the regulatory roles of ncRNAs, is a worthwhile research focus. A role of ICIs is Tregs markers and triggers for Treg-mediated inhibitory effects. Thus, it is also worthwhile to explicate how ncRNAs regulate these effector molecules.

In conclusion, the elaborate molecular mechanisms of how ncRNAs affect the differentiation and regulation of Tregs in various cancers remain enigmatic. Further functional studies on Tregs will not only aid our understanding of the role of ncRNAs in cancer immune responses and tumor immunotherapy but also develop Tregs as cancer immunotherapy targets.

## Author contributions

YM wrote this manuscript. HP designed the research. HW gathered information and performed literature retrieval. YL draw the figures. XX performed the proofreading and revise the manuscript. All authors contributed to the article and approved the submitted version.
